# Experiences of Transgender People Reviewing Their Electronic Health Records, a Qualitative Study

**DOI:** 10.1007/s11606-022-07671-6

**Published:** 2022-05-31

**Authors:** Ash B. Alpert, Jamie E. Mehringer, Sunshine J. Orta, Emile Redwood, Tresne Hernandez, Lexis Rivers, Charlie Manzano, Roman Ruddick, Spencer Adams, Catherine Cerulli, Don Operario, Jennifer J. Griggs

**Affiliations:** 1 Department of Health Services, Policy & Practice, Center for Gerontology and Healthcare Research, Brown University School of Public Health, Box G-S121-6, Providence, RI 02903 USA; 2 Department of Public Health Sciences, University of Rochester Medical Center, Rochester, NY USA; 3 Department of Pediatrics, University of Rochester Medical Center, Rochester, NY USA; 4 Trillium Health, Rochester, NY USA; 5 Department of Obstetrics and Gynecology, New York University Langone Health, New York, NY USA; 6 School of Medicine and Dentistry, University of Rochester, Rochester, NY USA; 7 Transgender Cancer Patient Project, Ashland, OR USA; 8 Western Michigan University, Kalamazoo, MI USA; 9 Department of Psychiatry, University of Rochester Medical Center, Rochester, NY USA; 10 The Susan B. Anthony Center, Rochester, NY USA; 11 Department of Behavior and Social Sciences, Brown University School of Public Health, Providence, RI USA; 12 Division of Hematology and Oncology, Department of Internal Medicine, University of Michigan, Ann Arbor, MI USA; 13 Institute for Healthcare Policy and Innovation, University of Michigan, Ann Arbor, MI USA

**Keywords:** transgender persons, electronic health records, patient-clinician communication, social stigma, qualitative research

## Abstract

**Background:**

The 21st Century Cures Act and the OpenNotes movement have brought patients immediate access to their electronic health records (EHRs). The experiences of marginalized people, including transgender people, accessing and reviewing their EHRs could inform documentation guidelines to improve patient-clinician rapport and reduce harm.

**Objective:**

To investigate the experiences of transgender people reviewing EHRs.

**Design:**

Qualitative study using community-engaged research and an interpretive description methodology. Participants were recruited via social media, snowball sampling was employed, and purposive sampling was used to ensure diversity in terms of age, race/ethnicity, and other factors. In focus groups, participants were asked to discuss their experiences reviewing their EHRs and, for those participants who were clinicians, their experiences reviewing other clinicians’ documentation.

**Participants:**

Thirty transgender adults aged 20 to 67 years, including 10 clinicians.

Approach: Digital audio-recordings of focus groups were transcribed verbatim. Content was analyzed to identify emerging essential elements and analysis was continued until no new themes emerged (i.e., saturation).

**Key Results:**

Four themes were noted. (1) Using the wrong name, pronoun, or gender marker for patients is common in the EHR, erodes trust, and causes trauma. (2) Various aspects of clinicians’ notes contradict, blame, or stigmatize patients, across multiple axes of oppression. (3) Limitations of EHR capabilities create barriers to quality care. (4) Certain medical customs set the stage for marginalizing, objectifying, and pathologizing transgender people.

**Conclusions:**

Transgender people experience harm via various aspects of EHR documentation, suggesting that changes must be made to improve patient-clinician relationships and reduce ill-effects for patients.

**Supplementary Information:**

The online version contains supplementary material available at 10.1007/s11606-022-07671-6.

## INTRODUCTION

Effective patient-clinician communication is essential to the delivery of high-quality care. Clinician practices that enhance meaningful connections with patients include close listening, responding to emotional cues, considering patients’ life circumstances, and centering patient priorities.^1^ Recently, the 21st Century Cures Act and the international OpenNotes^2^ movement have brought patients immediate digital access to clinician documentation in the electronic health record (EHR) and have expanded patient-clinician communication to the written form and the digital realm. An opportunity thus exists to explore documentation best practices to sustain and deepen patient-clinician relationships.

OpenNotes has many benefits, including increased rapport with patients and improved patient care.^3, 4^ However, medical documentation is also a means by which clinicians express approval or disapproval of patients’ behavior, question patient credibility, reinforce medical authority, and stereotype patients based on race, class, and gender.^5^ Stigmatizing medical documentation also influences other collaborating clinicians to adopt negative attitudes toward patients and to manage pain less aggressively.^6^

Transgender people may be particularly vulnerable to the harms of medical documentation given the pervasiveness of stigma, blaming, discrimination, and microaggressions in their clinical encounters.^7–10^ See Table 1 for definitions of stigma, microaggressions, and other key terminology. These experiences contribute to minority stress—the stress caused by chronic stigma and discrimination resulting in psychologic and physical sequelae.^11^ Such harms may be amplified for patients at the intersection of multiple marginalized identities, resulting in more extreme barriers to care and health disparities.^12^ EHRs of transgender people contain *misgendering*^13^*—*use of the wrong name, gender marker, or pronouns for patients. Use of the wrong name in EHR documentation is replicated on identification bands and rosters in inpatient settings and noted by clinicians and patients to contribute to misgendering.^14–16^ International Classification of Diseases (ICD) codes contain pathologizing or stigmatizing language, and EHR and billing workflows, such as requiring diagnoses for medication prescription to be included on a “problem list,” further pathologize patients﻿.^11, 17, 18^

**Table 1:** Definitions

Microaggressions	Indirect, or even unintentional, subtle discrimination or stigmatization.^9,23^
Stigma	The situation in which individuals or members of a social group are not allowed full social acceptance and instead are subject to othering. Stigma facilitates blaming, or language that suggests patients cause their own distress or discrimination.^8^
Intersectionality	The ways in which the interconnected status of social categories such as race and gender apply to marginalized groups and result in disproportionate health inequities.^10^
Misgendering	Referring to patients in a way that contradicts their gender. This can include use of the wrong name, pronouns, or gender marker.^24^
Dead names	The name given at birth for a transgender person, which is no longer used.^25^

However, to our knowledge, transgender peoples’ experiences reading their medical documentation and the impacts of stigmatizing or invalidating aspects of the EHR are unstudied. Patient perspectives are essential in understanding the effects of medical documentation on the reader and in suggesting ways to limit barriers to healthcare and the exacerbation of health inequities. Thus, we explored transgender patients’ experiences reviewing EHRs.

## METHODS

### Study Design

This qualitative study was conducted from September 2020 to August 2021 using community-engaged research^22, 23^ in collaboration with a community advisory board (CAB). The CAB is comprised of four transgender people in their 20s and 30s, three of whom are White and one of whom is Black. Two CAB members are non-binary, one is a man, and one is a woman. The CAB members have worked with the first author on transgender health research for the last 3 years and are paid $50/meeting. They assisted with developing the focus group guide, recruiting participants, and presenting the data and are included as co-authors on the manuscript.^22, 23^ An interpretive descriptive research design was used, a methodology created to develop knowledge about human health and illness experiences and uniquely suited to generate hypotheses applicable in clinical contexts.^24^

### Participants and Recruitment

Participants were purposively sampled to ensure diversity in age, geographic location, race/ethnicity, and gender. To be eligible, participants had to be 18 years of age or older, transgender (i.e., anyone who was not cisgender including non-binary, genderqueer, agender, and genderfluid people), English-speaking, and residing in North America. They were recruited via email announcements and postings on social media. We used snowball sampling to leverage social networks and, in particular, to reach transgender people of color. Transgender clinicians were recruited through the investigators’ professional networks. Thirty-seven people met inclusion criteria and purposive sampling goals and 30 chose to participate. This article adheres to the consolidated criteria for reporting qualitative research (COREQ).^25^ The University of Rochester Institutional Review Board approved this study. Participants provided verbal informed consent after reviewing risks and benefits of the study and being given the opportunity to ask questions. Written consent was not obtained, as unwanted disclosure of participants’ identities could have caused considerable harm. Following focus groups, each participant received a $25 gift card.

### Data Collection

Researchers A.A. and S.O. facilitated seven focus groups using a semi-structured interview guide and secure Zoom video conference software.^26^ Focus groups were used to allow participants to share experiences and theorize together. Specific groups were held for transgender people of color, clinicians, people who had received specialty care, and transfeminine participants with the hope of creating safer spaces for participants to speak openly. The guide was developed to be neutral (e.g., asking about positive and negative experiences), informed by conversations with the CAB, and iteratively revised based on feedback from participants. See Supplemental Appendix for the focus group guides. Focus group questions centered on participants’ experiences seeking healthcare, reviewing their medical records, and—for those who were clinicians—reviewing other clinicians’ documentation about transgender people. Participants also completed a demographics survey in REDCap.^27, 28^

### Data Analysis

Focus groups were digitally audio recorded, transcribed verbatim, and entered into ATLAS.ti Software.^29^ Two investigators (A.A. and J.M.) developed a codebook by independently reviewing and open coding the first transcript and then comparing, refining, and collapsing codes to create an initial codebook. They then coded each focus group transcript iteratively: independently double-coding, mutually reviewing their coding schemas, and reconciling each coding discrepancy as well as refining the codebook. They then summarized and examined the contents of each code for patterns and themes and continued until no new themes emerged (i.e., saturation). A third investigator (T.H.) then reviewed the coded transcripts and resultant themes and provided input. Emerging themes were also reviewed with the CAB, study participants, uninvolved clinically oriented researchers, and coinvestigators for member-checking and peer debriefing.

## RESULTS

The 30 participants had a median age of 31 years (range 20 to 67). Approximately half of participants were people of color and half were White and non-Latinx. Participants were geographically diverse, residing across the USA and one in Canada. Approximately half made less than $40,000 per year and a quarter less than $20,000. Most had private insurance. See Table 2 for participant characteristics. The analysis resulted in four major themes. (1) Misgendering is common in the EHR, erodes trust, and causes trauma. (2) Various aspects of clinicians’ notes contradict, blame, or stigmatize patients, often in a way that is intersectional for people who belong to more than one marginalized group. (3) Limitations to the EHR capabilities create barriers to quality care. (4) Various qualities of the medical system set the stage for marginalizing, objectifying, and pathologizing transgender people. We present these themes alongside illustrative quotations. With each quote, we also include age, race, and gender as described by participants.

**Table 2:** Participant Characteristics, *N* = 30

Age, mean (range)	31 (20–67)
Clinician	10 (33%)
Race/ethnicity	
Indigenous	3 (10)
Black	7 (23)
Asian	2 (7)
White	22 (73)
Latinx	4 (13)
Gender	
Non-binary/gender fluid/genderqueer/agender	21 (70)
Man	4 (13)
Woman	3 (10)
Does not identify with gender	1 (3)
Transgender man	1 (3)
Sexual orientation	
Queer/pansexual	22 (73)
Bisexual	6 (20)
Gay/lesbian	5 (17)
Heterosexual	1 (3)
Asexual	3 (10)
Something else	4 (13)
Choose not to answer	1 (3)
Income	
Less than $20,000	8 (27)
$20,000–39,999	6 (20)
$40,000–59,999	3 (10)
$60,000–79,999	2 (7)
>$80,000	5 (17)
Missing	4 (13)
Insurance	
Private	19 (63)
Public	
Medicaid	6 (20)
Medicare	4 (13)
Uninsured	2 (7)
Missing	1 (3)
Geographic region	
Western United States	8 (27)
Southwest United States	1 (3)
Midwestern United States	3 (10)
Southeastern United States	3 (10)
Northeastern United States	15 (50)
Canada	1 (3)

Values documented at *n* (%) unless otherwise noted. Participants were asked to “choose all that apply.” Thus, column values may not total *n*=30 nor percentages 100%.

### Theme 1: Misgendering Is Common in the EHR, Erodes Trust, and Causes Trauma

All participants with access to their EHR reported being misgendered in their medical records. These experiences elicited shame, embarrassment, frustration, anger, disappointment, and a sense of emotional exhaustion. Many participants noted that misgendering in the EHR was common even by clinicians with whom they had built rapport, including clinicians who had used their name and pronouns correctly during the clinical encounter.

[My clinician] calls me a “woman” even though we’ve had conversations, so it’s kind of triggering…I have a really good relationship with most of my doctors. They know me. … They know I’ve changed pronouns over time, they know, and it’s like, Really?

(White, non-binary, 55)

When providers used correct language in front of the participant, yet misgendered them in documentation, participants described losing trust—not only in the individual provider, but also in the entire field.

…All of my psychiatric [clinicians] misgender me in every single one of their notes despite having my pronouns listed at the top, which really erodes my trust in like that provider, but also every other provider in that field.

(White, non-binary, trans-feminine, genderqueer, gender fluid, 23)

The contrast between being respectfully addressed by a clinician in-person and seeing signs and symbols of allyship on display in the clinic, while being misgendered in documentation, led many participants to feel as though their clinicians were being disingenuous or performative.

There’s like stickers there that are like LGBTQ affirming, blah blah… [Yet] they both misgendered me in their goddamn notes!

(White, non-binary, 23)

Yeah, because as we know performance isn’t, it’s not genuine. Right?

(Black, man, 38, clinician)

In addition, participants described the ways that misgendering influenced their desire to share information with clinicians or request changes to medication.

... The fact that I use they/them pronouns never came up [with my clinician and]…he would narrate to me exactly what he’s typing …And so naturally it would be like, “She is doing well, blah blah blah”…These things [made] … such a difference in terms of how much [I shared]…I was just always trying to leave as fast as possible. Even [when] I knew I needed some changes in medication….

(White, genderqueer, 24)

Participants suggested that pronouns, name, and gender identity be used consistently throughout medical documentation.

Use the pronouns that the patient told you to use…it’s not enough to use it when you’re talking to the patient … you need to use it in your notes. …The same thing goes for the gender identity and the language that they asked you to use. It’s very frustrating to see notes like, “She wants to be called he.” And the rest of it is like, “She said.” It’s like, you almost got it. You’re almost there. But you have to keep going.

(White, non-binary, genderqueer, gender fluid, transmasculine, 31, clinician)

To counter these instances of misgendering in the EHR, one clinician participant described using medical documentation to advocate for the use of the right name and pronouns for a patient.

I wrote the note…and I really emphasized this patient’s name and his pronouns.

(Black, non-binary, genderqueer, 28, clinician)

### Theme 2: Various Aspects of Clinicians’ Notes Contradict, Blame, or Stigmatize Patients, Often in a Way That Is Intersectional for People Who Belong to More than One Marginalized Group

In addition to misgendering, participants described other ways in which clinicians’ documentation implied that their gender was invalid. Participants reported that clinicians placed quotes around their pronouns or name, or included terms such as “identifies as” or “preferred” to suggest doubt or communicate transphobia.

The provider put everything [the patient] said in quotes that related to their gender…So that made me feel…like the provider didn’t believe that person.

(Latinx, White, non-binary, 30, clinician)

My pronouns, like anybody’s pronouns, they’re not, they’re not a preference. They’re non-negotiable…

(Black, man, 38, clinician)

A friend of mine had an EHR that said “Jessica is a male that prefers to dress as and be referred to as a female.” And then proceeded to “he/him” her the whole way through. And none of that, none of that is affirming.

(Latinx, trans man, 34, clinician)

I don’t want to see “identifies as” and I don’t want to see “preferred pronouns.” Those are both microaggressions and they’re really annoying because part of being respected is not having my gender cast into metaphysical doubt.…I am nonbinary; I don’t identify as nonbinary.

(White, non-binary, 26)

One participant also described feeling uncomfortable when their symptoms were described using gendered language not reflective of the language they had reported.

…It seems to be talked about in a very like cis women-centric way rather than how I talked about it. I would describe the pain, like specifically where and how it felt, and then they would put “period pain.”

(White, non-binary, 25)

Multiple participants described stigma in documentation across various characteristics such as body size, mental health, neurodiversity, ability, and race. Additionally, participants described the ways that documentation of other marginalized identities seemed to reinforce and increase stigma regarding transgender identity.

…When somebody puts a mental health diagnosis next to the person’s gender identity, they’re trying to communicate something about the validity of that gender identity and it’s not usually a nice thing… like “so and so is a 58-year-old bipolar transgender woman.”

(White, non-binary, genderqueer, gender fluid, transmasculine, 31, clinician)

One participant who worked in a hospital explained that clinicians used racial stereotypes to label patients who advocated for being gendered correctly as “aggressive” or “hostile” and explained that such language was then repeated throughout EHR and led to poorer care.

[In the EHR]… those details that people added in the notes section can definitely get used against [the patients]… especially if you’re a person of color and you’re trying to be enforcing pronouns, you’ll usually get labeled as “hostile” … Usually when [patients] are just trying to enforce their own boundaries… and then that establishes a pattern in your medical record that then is used to treat you poorly. Or to not be listening to what you’re saying…

(Xicanx — mixed native, Latinx, and White, non-binary, 30)

### Theme 3: Limitations to the EHR Capabilities Create Barriers to Quality Care

Participants noted limitations in the EHR’s ability to correctly interpret laboratory tests for transgender people, resulting in persistently abnormal values, causing stress and uncertainty.

When I get labs done, they have me as a female for my lab levels, and so they’re always a little bit off, and it freaks me out and I’m like, Is this normal?

(White, man, 22)

EHRs also lack the ability to deftly manage gender-related data such as name and gender marker for the simultaneous purposes of billing, insurance coverage, and clinical encounters, resulting in people being persistently misgendered in their EHR without a means to change this. Occasionally, outdated information about name and sex-assigned-at-birth was electronically propagated or transferred between systems causing participants to be re-confronted with so-called dead names, or names they no longer use, or old gender markers despite changing these in the EHR previously.

…The first time I went to pick up prescriptions from [a new clinician], they had my dead name on the medication that I picked up – the system of [a clinician] who had never known me by any other name other than my legally changed name right? And it was like a really big shock to see that…kind of scary, actually.

(White, non-binary, 26)

Participants also suggested that EHR systems were often unable to correctly capture their gender or pronouns; this was especially true for non-binary people with they/them pronouns. This resulted in non-binary people having EHR documentation that did not apply to them.

[In my chart] there was a medical alert that stated, “transgender patient, FTM.” I’m a nonbinary person, though I am assigned female at birth, however I don’t identify or relate to the frame FTM. When I said that, the only explanation I got was the only options were FTM or MTF.

(Black, non-binary, 28)

…Any time I bring up my gender, it’s like this clusterfuck of their systems are breaking; they just cannot figure out how to categorize me [in the EHR].

(Asian, non-binary, agender, 21)

Clinicians described being unable to get certain procedures (e.g., cervical cancer screening) covered by insurance without changing a patient’s gender marker in the EHR, thus setting the stage for trauma.

…If you’re caring for someone who is transmasculine and they’re getting…a Pap smear, you have to change …someone’s gender identity in order for insurance to cover it. But if someone had access to their medical record…that can be traumatizing.

(Black, non-binary, genderqueer, 28, clinician)

Participants suggested clinicians ask patients what they want included in the EHR.

… It would probably be helpful for the doctor to explicitly say… “if you’re accessing your notes, please let me know if there’s anything that you’d rather I change…”

(White, non-binary, genderqueer, gender fluid, 25)

I personally appreciate that …my physician…asked me “which diagnosis would you like me to put so that you can get your testosterone covered?” …I would say [physicians should] just ask what the [patient] wants.

(Asian, non-binary, agender, 21)

### Theme 4: Various Qualities of the Medical System Set the Stage for Marginalizing, Objectifying, and Pathologizing Transgender People

Participants discussed the ways that various aspects of medical culture may run counter to creating documentation that is non-stigmatizing and reflects patients’ identities. Multiple participants, including those who were clinicians, commented on the one-sentence summary, or “one-liner”—the sentence that begins clinical assessments and summarizes potentially relevant patient characteristics and symptoms—which often includes age, gender and/or sex-assigned-at-birth, and race. Some clinicians described changes they had made to the one-sentence summary to be more patient-centered and humanizing and to ensure that specific demographic categories were not used to limit the differential of other clinicians.

[I try] to humanize the experience in general when I’m writing notes. Like the standard is just to be like, “50-year-old male blah blah blah” and so I say their name….I imagine if someone were to read this note it doesn’t just feel like they’re just some—almost like an object—but an actual human being. … I’ve had a lot of discussions with colleagues about …moving away from identifying [race] in the note… [that] can put tunnel vision on people’s views of what’s going on, which is harmful to the patient if [clinicians are not]… thinking of a full differential.

(Black, non-binary, genderqueer, 28, clinician)

Participants similarly described a preference for gender and sex-assigned-at-birth to be left out of one-sentence summaries.

...When we discuss gender, I think we should discuss gender and not discuss sex…

(Black, non-binary, 28)

I like it when some notes just introduce me as a 25-year-old, and that’s it. Like [patient] is a 25-year-old and then they’ll just go into whatever we talked about. I like that more than gender introduced at all.

(White, non-binary, 25)

Responding to that, another participant said:

… or putting sex assigned at birth as if it’s an indicator of the anatomy that I have right now or that it’s really indicative of anything…Because it often isn’t…

(White, man, 22)

Diagnostic codes used for billing could also be stigmatizing or incongruent with patients’ sense of themselves. For example, one participant acknowledged that diagnostic codes are necessary in the current system to obtain insurance coverage for medications such as HIV pre-exposure prophylaxis but suggested that they also cause harm.

“N90.5 vaginal atrophy” for estrace cream. I don’t love that... Or for PrEP, “high risk homosexual behavior,” don’t love that. I recognize that [clinicians] need to do that for coding and billing, so I guess I accept it because, ultimately, I want something also. But…the phrasing…does do a lot of harm.

(White, non-binary, 32, clinician)

Participants also noted that transgender identities were pathologized in EHRs via diagnostic codes.

I remember … seeing I think it was like “history of transexualized surgery.”…It felt stigmatizing and medically unhelpful at the same time. You just said that there’s something transexual about me. But there is nothing medically helpful in there.

(White, non-binary, genderqueer, gender fluid, transmasculine, 31, clinician)

In particular, some participants described concerns with the diagnosis of “gender dysphoria,” particularly as a prerequisite for specific types of care.

I don’t identify with the diagnosis of gender dysphoria …When somebody tells me I have gender dysphoria I feel like they’re putting the blame on me. And …if I was born in a different society where you don’t assume my gender, maybe I would not have needed to go on hormones. And for me I blame the rest of the world for it.

(Black, doesn’t believe in gender, 29)

## DISCUSSION

Current EHR systems and clinician documentation practices can cause iatrogenic trauma to transgender individuals through repeated misgendering, invalidating, and stigmatizing, which can erode trust in clinicians and the medical field, impede delivery of quality care, and further exacerbate health inequities. Additionally, these stigmatizing aspects of documentation can contribute to minority stress and lead to adverse health sequelae for patients.^11^ This harm is likely compounded for people who belong to more than one marginalized group. Limitations in EHR capabilities result in further trauma as well as barriers to quality care. Deeply entrenched aspects of medical systems, such as one-sentence summaries and diagnostic coding, also set the stage for othering and pathologizing transgender people. The OpenNotes movement provides an opportunity for clinicians, administrators, and policymakers to learn from the insights of patients and shift medical documentation.

Previous research has demonstrated that physician documentation contains misgendering^14^ and stigma^5^ that both reflect and perpetuate negative attitudes toward patients and subpar care. Our work expands upon these findings by identifying ways in which clinician notes, which often contain misgendering and invalidating language, can cause harm to the patients reviewing them.

Although our study is novel and provides rich, vivid accounts of the experiences of transgender people, allowing readers to gain insight into the impact of stigmatizing medical documentation, it has several limitations. The sample was exclusively adult, predominantly young adult to middle-aged, non-binary, gender fluid, genderqueer, and agender people, and living exclusively in North America. Focus groups also have a number of limitations including that more vocal participants can dominate discussions such that people with dissenting opinions may be less likely to speak up. Also, focus groups do not allow for direct observation or deep investigation of individual experiences. Future studies should test training for clinicians based on patient-generated documentation recommendations and EHR-based interventions, which would make shifts in practice more feasible. Research to assess normal laboratory ranges based on objective measures such as anatomy or hormone levels is also called for.

Based on our findings, we recommend that healthcare workers document the gender, name, and pronouns per the patient without quotation marks as well as consistently using this information throughout notes. Additionally, we advise avoiding (1) the word “preferred” when writing patients’ names and pronouns, (2) the phrase “identifies as” when recording patients’ genders or pronouns, and (3) medically irrelevant mention of the patient’s sex-assigned-at-birth. Our findings also suggest the importance of removing pathologizing International Classification of Diseases (ICD) codes from notes, avoiding irrelevant information in the one-sentence summary, avoiding language which communicates judgment or blame, and proactively discussing documentation with patients. See Table 3 for a summary of documentation recommendations.

**Table 3:** Recommendations for Documentation

Use the correct pronouns consistently throughout all documentation unless otherwise requested by patient.	
Refer to patients by the correct (i.e., the ones they have specified) name consistently throughout all documentation unless otherwise requested by patient.	
Avoid the use of language that may inadvertently question the legitimacy of a patient’s gender. • Document gender, name, and pronouns without quotation marks around them. • Document name and pronouns without the term “preferred.” • Document gender without the phrase “identifies as.”	
Avoid medically irrelevant mention of sex-assigned-at-birth	
Document the patient’s stated gender. Avoid terms such as “male-to-female,” “female-to-male,” “FTM,” or “MTF” unless patient specifies identifying with one of these terms.	
Remove stigmatizing International Classification of Diseases codes and terminology from notes. Examples include “Gender Identity Disorder” and “High risk homosexual behavior.”	
Proactively discuss documentation with patients. If difficult to avoid stigmatizing diagnostic terms, discuss with patients in advance of documenting and engage in shared decision-making based on risks and benefits.	
Include only clinically relevant information in the one-liner.	
Avoid language in documentation that communicates judgment or blame, for example words such as “hostile” and “aggressive.”	

We suggest that these guidelines be incorporated into medical, nursing, and allied health professional training and training of clerical staff, such as the eQuality curriculum.^21^ Such training should also include information about the adverse effects of stigmatizing or invalidating documentation when patients review their EHR as well as the impact of stigmatizing language on other clinicians (e.g., stigma “contagion” within health service settings).^30, 31^ The landscape of thinking in regard to incorporation of gender, pronouns, sex assignment, and anatomy data into EHR documentation is evolving, with the field moving toward greater data accuracy and visibility and inclusion of transgender people into system design.^17, 19, 20^ Incorporating patient perspectives into recent recommendations to develop national standards for EHR documentation may ensure these changes are patient-centered, non-stigmatizing, and implemented widely. Health systems could hold clinicians and staff accountable by instituting peer review of medical documentation and including these peer reviews in decisions about promotion and tenure along with other metrics of clinical care. In addition, changes should be made to EHR systems to allow for customization such that (1) labs are interpreted correctly based on hormonal milieu and anatomy rather than based on a sex or gender label and (2) clinicians can override or correct lab interpretation flags. Revisions to ICD codes to allow for less stigmatizing language are also necessary. OpenNotes provides us an opportunity to decrease stigmatization in the EHR and our systems overall to improve patient-clinician relationships and the health of all patients.

## Supplementary Information

ESM 1 (DOCX 18 kb)
