# Supplementary material for: Experiences of Transgender People Reviewing Their Electronic Health Records, a Qualitative Study
Source: J Gen Intern Med. 2022 May 31;38(4):970–7. doi: 10.1007/s11606-022-07671-6 (PMC10039220; doi:10.1007/s11606-022-07671-6)
Supplement: Supplementary file 1 — (DOCX 18 kb) [file 11606_2022_7671_MOESM1_ESM.docx]

**Focus Group Script for Non-Clinicians**

Thank you for participating in this focus group. The purpose is to hear about your experiences with clinicians related to being transgender. It is becoming more common for patients to be reading their own medical records and we want to understand what that’s like for trans folks.

For today, you have all chosen a made-up name. We will do introductions so that everyone knows everyone else’s made-up name and pronouns. Please only refer to other people in the group by their made-up name even if you also know their real name and please use people’s correct pronouns.

We are going to start with introductions. Then I will begin asking questions and people can speak when they are ready. Please allow each person to finish speaking before starting to talk; speak respectfully to other people in the group (no racism or other isms will be tolerated in the group); and if you’ve already spoken, ensure that other people in the group have had the opportunity to speak before speaking again. Lastly, we ask that you not share the stories of the people who were here today. We will be recording this conversation and will use it to help us understand how to assess quality of care for transgender people using medical records.

Does anyone have any other ground rules to add to these?

Okay, we will get started.

1. As a transgender person seeking healthcare for yourself, have you had any positive or negative experiences with clinicians that stand out? If so, what were they? What details do you remember about the experience if any? For example, was there something that was said or done in the room? Something you overheard? Were there things that you picked up that weren’t what the doctor actually said but a vibe or something in their facial expression or tone of voice?

3. Have you seen any part of your medical record? If so, as a trans person, what’s that experience been like for you?

4. Was there anything that your clinician documented about your gender that surprised you?

6. How have you noticed clinicians documenting about gender? What, if any, words or phrases stand out as stigmatizing or not stigmatizing, affirming or not affirming?

7. Do you have any suggestions or advice for clinicians when it comes to documenting about a trans patient?

8. How have you noticed clinicians document about other aspects of identity (such as race, ethnicity, religion, class, age, size, ability) in the medical record or otherwise?

9. How do you think clinicians perceive transgender people? What makes you think that?

**Focus Group Script for Clinicians**

Thank you for participating in this focus group. The purpose is to hear about your experiences with clinicians related to being transgender and your experiences witnessing other clinicians interact with transgender people. It is becoming more common for patients to be reading their own medical records and we want to understand what that’s like for trans folks.

For today, you have all chosen a made-up name. We will do introductions so that everyone knows everyone else’s made-up name and pronouns. Please only refer to other people in the group by their made-up name even if you also know their real name and please use people’s correct pronouns.

We are going to start with introductions. Then I will begin asking questions and people can speak when they are ready. Please allow each person to finish speaking before starting to talk; speak respectfully to other people in the group (no racism or other isms will be tolerated in the group); and if you’ve already spoken, ensure that other people in the group have had the opportunity to speak before speaking again. Lastly, we ask that you not share the stories of the people who were here today. We will be recording this conversation and will use it to help us understand how to assess quality of care for transgender people using medical records.

Does anyone have any other ground rules to add to these?

Okay, we will get started.

1. As a transgender person seeking healthcare for yourself, have you had any positive or negative experiences with clinicians that stand out? If so, what were they? What details do you remember about the experience if any? For example, was there something that was said or done in the room? Something you overheard? Were there things that you picked up that weren’t what the doctor actually said but a vibe or something in their facial expression or tone of voice?

2. As a clinician, have you witnessed any interactions between other clinicians and transgender people that stand out? If so, what were they?

3. Have you seen any part of your medical record? If so, as a trans person, what’s that experience been like for you?

4. Was there anything that your clinician documented about your gender that surprised you?

5. Have you seen the medical records of other transgender people? If so, what aspects stand out?

6. How have you noticed clinicians documenting about gender? What, if any, words or phrases stand out as stigmatizing or not stigmatizing, affirming or not affirming?

7. Do you have any suggestions or advice for clinicians when it comes to documenting about a trans patient?

8. How have you noticed clinician document about other aspects of identity (such as race, ethnicity, religion, class, age, size, ability) in the medical record or otherwise?

9. How do you think clinicians perceive transgender people? What makes you think that?
